# Successful Transplantation of a Split Crossed Fused Ectopic Kidney into a Patient with End-Stage Renal Disease

**DOI:** 10.1155/2010/383972

**Published:** 2010-03-25

**Authors:** Kristin L. Mekeel, Shane M. Daley, Paul E. Andrews, Adyr A. Moss, R. L. Heilman, Marek J. Mazur, Harini A. Chakkera, Khalid Hamawi, David C. Mulligan, K. Sudhakar Reddy

**Affiliations:** ^1^Division of Transplant, Hepatobiliary and Pancreatic Surgery, Mayo Clinic, Phoenix, AZ 85054, USA; ^2^Departmen of Urology, Mayo Clinic, Phoenix, AZ 85054, USA; ^3^Division of Transplant Nephrology, Mayo Clinic, Phoenix, AZ 85054, USA

## Abstract

Potential donors with congenital renal anomalies but normal renal function are often overlooked because of a possible increase in technical difficulty and complications associated with the surgery. However, as the waiting list for a deceased donor kidney transplant continues to grow, it is important to consider these kidneys for potential transplant. This paper describes the procurement of a crossed fused ectopic kidney, and subsequent parenchymal transection prior to transplantation as part of a combined simultaneous kidney pancreas transplant. The transplant was uncomplicated, and the graft had immediate function. The patient is now two years from transplant with excellent function.

## 1. Introduction

Historically, kidneys with congenital abnormalities were often discarded because of the perceived risk of technical complications. However, as the waiting times for kidney transplants continue to increase, transplant centers have become more aggressive at using select kidneys with congenital abnormalities. Horseshoe kidneys are now routinely used for transplantation, either as a single or split graft. Renal ectopia describes the failure of the kidney to ascend and cross the midline during development, resulting in the ipsilateral position of both kidneys. In many cases they remain fused. Although these kidneys have an increased rate of vascular and ureteral anatomical anomalies, there are select reports of use in renal transplantation. This is the first case report of the successful transplantation of a split crossed fused kidney for a combined simultaneous kidney pancreas transplantation.

## 2. Case Report

The transplant recipient is a 58-year-old female who developed type I diabetes mellitus at 6 months old and used an insulin pump for blood sugar control. She developed end organ damage including renal failure but was not yet dialysis dependent. She also suffered from retinopathy and peripheral neuropathy. She did not have hypoglemic unawareness and had good blood sugar control with her insulin pump. The remainder of her medical history included hypertension, hyperlipidemia, and hypothyroidism. She had remote hysterectomy and appendectomy. She underwent a full evaluation for kidney and pancreas transplantation without significant findings. She was approved and listed for combined simultaneous kidney pancreas transplantation. At the time of transplantation her panel of reactive antigen (PRA) was 0% and there were no identifiable HLA specific antibodies. Hr BMI was 26 at the time of transplantation.

**Figure 1:**
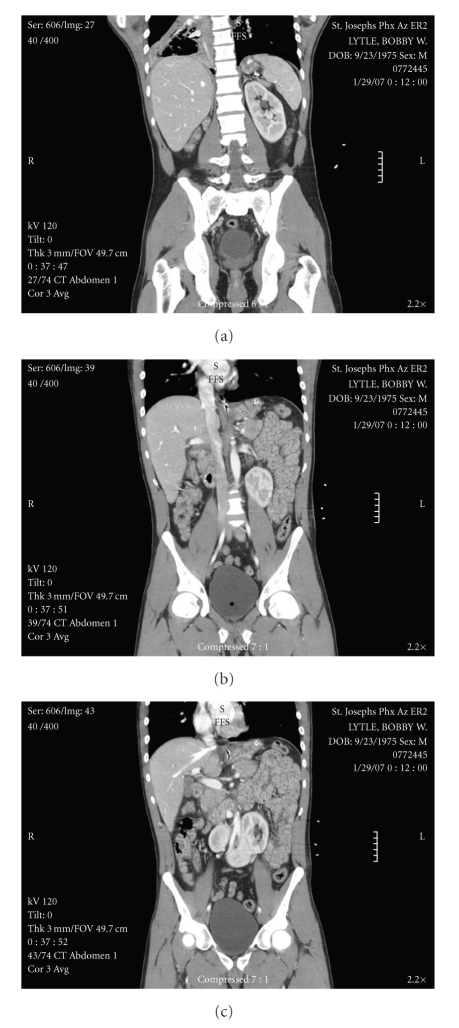
This is a series of coronal sections from a computed tomography (CT) scan of the abdomen and pelvis, done as a screening scan for abdominal trauma when the donor was admitted to the hospital. Image (a) shows the left kidney in a normal anatomic position. Image (b) shows the inferior fusion plane. Image (c) shows the right ectopic kidney, crossing the midline across the iliac vessels.

The donor was a 31-year-old male motorcyclist who collided with a concrete medium at highway speed. He was not wearing a helmet. The donor did not have significant past medical or surgical history, except for smoking cigarettes and occasional marijuana. There was no history of renal calculi or recurrent urinary tract infections. The donor family was not aware of his congenital renal anomaly and the patient did not have other congenital anomalies. A computed tomography (CT) scan of the abdomen done at the time of admission revealed an ectopic right kidney located in the pelvis and fused with the lower pole of the left kidney, compatible with crossed fused renal ectopia (Figure 1). There were no other significant findings. The patient was hemodynamically stable with good urine output the time of procurement. The serum creatinine peaked at 1.8 and was 1.4 at the time of procurement. There was mild elevation of blood glucose requiring sliding- scale insulin.

The procurement of the kidneys, pancreas, and liver was completed in the standard fashion, flushing with Custodial HTK (Odyssey) solution. The kidneys were procured en bloc, with a complete cava and aorta. The left kidney had a single artery, and the right had three arteries. The upper and mid polar arteries of the right kidney originated from the aorta, while the lower pole artery originated from the right common iliac artery. The mid and lower pole arteries were smaller, accessory arteries. There were single renal veins bilaterally, which drained into the inferior vena cava. The left ureter crossed the midline and entered the bladder in the normal anatomic position.

**Figure 2:**
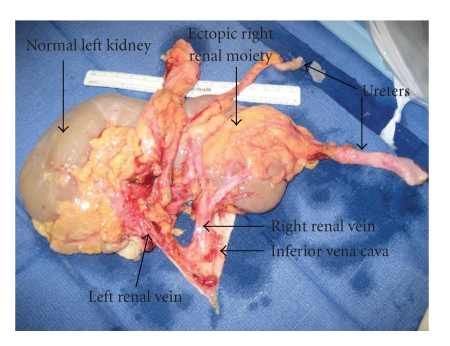
This intraoperative picture shows the renal mass on the back table, prior to transection.

**Figure 3:**
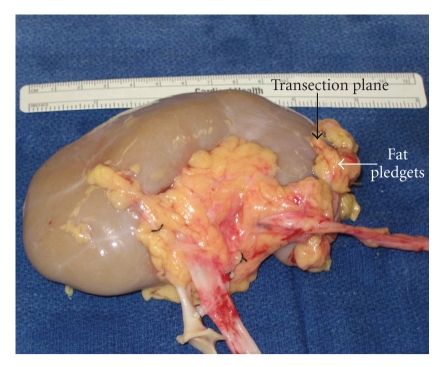
This intraoperative picture demonstrates the left kidney after transection, with the transected plane plegeted with fat and retention sutures.

The kidneys were transferred to our institution. The back table dissection and parenchymal transection was completed with the assistance of the urology service. Initially we had planned to split the kidneys and use each singly for transplant (Figure 2). However, the right kidney was abnormal in appearance; in particular it was very thin. The mid and lower pole arteries were very small and were not connected to the aortic patch, making them difficult to reimplant. We elected not use the renal mass en bloc, due to the complex arterial anatomy, including the number, caliber, and configuration. At that time the right kidney was split from the left, carefully transecting the isthmus using sharp dissection. A few vessels and what appeared to be a small calyx were suture ligated with Vicryl suture. Next, the transection plane was closed using interrupted 0 chromic mattress sutures. Pledgets of fat were used to prevent any cut through of the sutures through the parenchyma (Figure 3). Both the artery and vein were cleaned of surrounding neurolymphatic tissue.

The kidney and pancreas transplantation was then completed in the standard fashion. The kidney was anastamosed to the external iliac artery and vein on the left side. The kidney reperfused well, with minimal bleeding after reperfusion needing only bipolar coagulation for control. The pancreas graft was anastamosed to the right common iliac artery and vein and the donor duodenum to the recipient jejunum. Immunosupression included Campath induction, with Prograf, Cellcept, and a rapid steroid taper. The patient had immediate graft function and an unremarkable postoperative course. She was discharged on postoperative day seven. A protocol pancreatic biopsy at one year demonstrated Drachenberg grade III out V acute rejection. She was treated with thymoglobulin and Solumedrol and continued on steroids after discharge. She is now 2 years posttransplant with normal pancreatic (fasting blood sugar 86, c-peptide 2.1, hemoglobin A1C 5.4) and renal graft function (creatinine 1.0 and creatinine clearance 55).

## 3. Discussion

The continuing shortage of deceased donor kidneys has inspired transplant programs to search for innovative ways to increase the number of potential donor kidneys. It is now common for many centers to use en bloc pediatric kidneys, kidneys with acute renal failure, and two extended donor criteria kidneys in a single recipient [1–3]. Horseshoe kidneys are now routinely used as both dual and split single transplants, with results almost equivalent to deceased donor transplantation (83.3% graft survival at 5 year follow-up) [4]. The recent case reports describe the use of living donors and in situ split of the horseshoe kidney for transplantation [5].

Congenital renal fusion anomalies are seen in 1 in 250 autopsies, with horseshoe kidney being the most common anomaly present in 1 to 600–800 adults [5]. Renal ectopia has an incidence of 1/500–1200 and the ectopic kidney can be located from the pelvis (most common) to the thorax [6]. The combination of ectopia and fusion can be found at an incidence of 1/1000 [7] and is termed crossed fused renal ectopia (CFRE). Although there are 6 described variations of CFRE, the aberrant kidney is most often located inferior to the normally positioned kidney, and fusion occurs between the superior pole of the aberrant kidney to the inferior pole of the normal kidney [8]. Most patients remain asymptomatic, and the diagnosis is made at the time of autopsy or imaging done for another indication. However, ureteral obstruction, renal calculi, and an increased risk of neoplasm have all been described.

Kidney transplantation using donors with crossed renal ectopia has been described. There is a single case report of transplantation with a nonfused kidneys [9] and two case reports of crossed as a dual transplant [8, 10]. These cases are summarized in Table 1. Both of these reports describe the en bloc transplantation of both fused kidneys. This is the first report of splitting the crossed fused renal ectopia prior to transplantation as well as in combination with a simultaneous pancreas transplant. CFRE may be diagnosed preoperatively on routine imaging or intraoperatively at the time of donation. Donors with a history of renal calculi or recurrent urinary tract infections in combination of CRFE should be excluded because of the potential for postoperative obstructive complications.

Familiarity with the potential anatomic anomalies associated with CRFE is essential to prevent injury to the kidneys at the time of procurement. More than 70% of CRFE have multiple arteries and multiple veins are also common [10]. It is optimal for the kidneys to be procured en bloc, with consideration for transection done at the recipient transplant center. Greater than 90% of patients with CRFE have two ureters. Unlike with horseshoe kidneys where the ureters are located anteriorly in a normal anatomic position, ureters from the crossed fused ectopic kidney crosses over the midline to left side to enter in the bladder in a normal anatomic position [11]. Care must be taken to avoid ureteral transection, especially during the contra-lateral iliac dissection.

Investigation of the kidneys should be on the back table to consider for possible split. Obviously, obtaining two grafts is preferable to using one, but it may not be possible secondary to anatomic anomalies. Both the aberrant and normally positioned kidney can have variant arterial supply from the upper abdominal aorta, lower aorta, and iliac arteries [8]. If the number or size of the arteries is too complex for transplantation, splitting of the ectopic moiety (as in our case) or en bloc transplantation [10] is recommended. In our case, splitting of the ectopic kidney helped to facilitate transplantation of the normal kidney. Venous anatomy can also be complex. If multiple renal veins are encountered during routine transplantation, most surgeons routinely ligate smaller renal veins without affecting the outflow of the kidney. However, in these cases there may not be collateral venous drainage between the two kidneys and anastamosis of even smaller veins may be necessary to prevent venous hypertension.

Horseshoe kidneys and CFRE are separated by a tissue band, which ranges from a thin fibrous isthmus to thick functional parenchyma. The techniques used for renal transection in partial nephrectomies are applicable to the back table splitting the fused kidneys. This includes sharp transection followed by use of the tissue link or cautery for hemostasis, as well as direct locking suture ligature of the stump. In addition, the use of hemostatic glues and materials can be used to help attain hemostasis and prevent a urine leaks [12]. In this case, the isthmus was thin and a sharp transection followed interlocking suture closure of the defect in combination with peri-renal fat pledgets was sufficient to prevent both hemorrhage and urine leak. If the bridge of tissue is too thick to allow safe transection, the kidney can be transplanted as a single graft or en bloc.

The last consideration is that the CRFE kidney may be difficult to position due to the vascular reconstruction and increased renal mass. As pointed out by Bailey et al., opening of the peritoneum for positioning may be necessary if the transplant is completed through an extra-peritoneal approach [8]. In this paper, the kidney was placed intraperitoneal and secured behind the sigmoid colon to prevent torsion. Although the use of a living donor split horseshoe kidney has been reported in the literature [5], the complex vascular and arterial anatomy, as well as potential compromise of the donors remaining kidney, would make living donation treacherous.

## 4. Conclusion

Crossed fused renal ectopia should not be considered a contraindication to transplantation. However, the kidneys must be both procured and transplanted with careful attention to the anomalous vascular and ureteral anatomy, as well as graft placement to prevent torsion. If there are multiple vascular structures, or the transaction plane does not result in adequate nephron mass for both kidneys, consideration of transplanting the kidneys en bloc or resection the smaller moiety should be considered. Transplant surgeons should be familiar with these potential anatomic variations to ensure these grafts are not wasted. Long term graft survival does not appear to be limited in these otherwise healthy donor kidneys.

## Figures and Tables

**Table 1:** 

Case Reports	Bailey et al. [8]	Ojo et al. [10]
*Donor age/sexCOD*	36/MCHI	20/MMVA
*Diagnosis made*	Procurement	Pre-operative CT scan
*Description of Fusion*	Left sided with inferior ectopia	Right sided with inferior ectopia
*Arterial anatomy*	3 arteries originating from infra-renal aorta	3 arteries originating infra-renal aorta and at iliac bifurcation
*Arterial reconstruction*	Two separate anastamoses, one of the single dominant artery and the second a patch of the two smaller arteries	Donor aorta directly to recipient iliac
*Venous anatomy*	Two veins originating from IVC	Five veins originating from IVC
*Venous reconstruction*	Two separate anastamoses to recipient iliac vein, initially one vein was ligated but reopened with evidence of venous hypertension.	Donor IVC to recipient iliac vein
*Ureteral anatomy*	Two ureters, separate bladder anastamoses.	Two ureters, separate bladder anastamoses.
*Other information*	Peritoneum opened to facilitate positioning	
*Follow-up*	40 months, normal graft function	30 days, normal graft function

CHI=closed head injury, MVA=motor vehicle accident, CT=computed tomography, IVC=inferior vena cava.
